# Role of Artificial Intelligence in Identifying Vital Biomarkers with Greater Precision in Emergency Departments During Emerging Pandemics

**DOI:** 10.3390/ijms26020722

**Published:** 2025-01-16

**Authors:** Nicolás J. Garrido, Félix González-Martínez, Ana M. Torres, Pilar Blasco-Segura, Susana Losada, Adrián Plaza, Jorge Mateo

**Affiliations:** 1 Internal Medicine, Virgen de la Luz Hospital, 16002 Cuenca, Spain; 2 Expert Medical Analysis Group, Institute of Technology, University of Castilla-La Mancha, 16071 Cuenca, Spain; 3 Expert Medical Analysis Group, Instituto de Investigación Sanitaria de Castilla-La Mancha (IDISCAM), 45071 Toledo, Spain; 4 Department of Emergency Medicine, Virgen de la Luz Hospital, 16002 Cuenca, Spain; 5 Department of Pharmacy, General University Hospital, 46014 Valencia, Spain

**Keywords:** hospital mortality, emergency, biomarkers, machine learning, pandemics, predictive value of tests

## Abstract

The COVID-19 pandemic has accelerated advances in molecular biology and virology, enabling the identification of key biomarkers to differentiate between severe and mild cases. Furthermore, the use of artificial intelligence (AI) and machine learning (ML) to analyze large datasets has been crucial for rapidly identifying relevant biomarkers for disease prognosis, including COVID-19. This approach enhances diagnostics in emergency settings, allowing for more accurate and efficient patient management. This study demonstrates how machine learning algorithms in emergency departments can rapidly identify key biomarkers for the vital prognosis in an emerging pandemic using COVID-19 as an example by analyzing clinical, epidemiological, analytical, and radiological data. All consecutively admitted patients were included, and more than 89 variables were processed using the Random Forest (RF) algorithm. The RF model achieved the highest balanced accuracy at 92.61%. The biomarkers most predictive of mortality included procalcitonin (PCT), lactate dehydrogenase (LDH), and C-reactive protein (CRP). Additionally, the system highlighted the significance of interstitial infiltrates in chest X-rays and D-dimer levels. Our results demonstrate that RF is crucial in identifying critical biomarkers in emerging diseases, accelerating data analysis, and optimizing prognosis and personalized treatment, emphasizing the importance of PCT and LDH in high-risk patients.

## 1. Introduction

The COVID-19 pandemic has significantly transformed the clinical and immunological profiles of patients. Insights into the molecular composition of the virus have been crucial in identifying emerging variants, such as *Omicron* (*BA.2*, *BA.4*, *BA.5*), predicting their behavior, and implementing large-scale vaccination programs [1,2,3]. These vaccines have demonstrated high efficacy in reducing disease severity and mortality; however, challenges such as superinfections and allergic reactions persist. Although hypersensitivity reactions to these vaccines are rare, guidelines from organizations like the European Academy of Allergy and Clinical Immunology (EAACI) have provided valuable recommendations for their management [4,5].

During this period, established prediction tools for other diseases, including the Wells scale and the YEARS algorithm, have been applied to COVID-19 patients to evaluate their utility. Findings indicate that these models, originally developed for the general population, exhibit limitations when applied to COVID-19 cases, highlighting the necessity for disease-specific predictive tools [6,7,8].

Additionally, geospatial models have been utilized to estimate COVID-19 infection and mortality rates. These models have been effective in identifying high-risk conditions [9], though their accuracy could be enhanced by integrating additional factors, such as mortality in long-term care facilities and adherence to social distancing protocols.

Advancements in molecular biology and virology have enabled the identification of critical conditions associated with COVID-19, such as multisystem inflammatory syndrome in children (MIS-C), a serious complication with low mortality rates [9]. Alterations in immune cells, including T cells, B cells, and mast cells, have been linked to long-term symptoms in some patients, commonly referred to as prolonged COVID-19. The potential association between atopic diseases, such as asthma and rhinitis, and susceptibility to COVID-19 remains a topic of ongoing investigation.

These molecular biology advancements have been applied to establish correlations between specific molecules and severe COVID-19 outcomes. Studies have revealed a reduced diversity and increased clonal expansion of T-cell receptors in severe cases, particularly in patients under 55 years old, suggesting an impaired immune repertoire in this group [10,11]. Additionally, molecular biomarkers such as ferritin, D-dimer, C-reactive protein (CRP), and lactate dehydrogenase (LDH) have been identified as effective indicators for distinguishing mild from severe cases. Higher expression of certain genes, such as *MX1* and *AR*, has been associated with lower risk in women [12,13,14].

The understanding of these biomarkers has also deepened insights into the effects of both the infection and the vaccines developed. For instance, studies have examined the relationship between the Sputnik vaccine and autoimmune diseases, reporting neurological and hematological manifestations such as thrombosis and Guillain–Barré syndrome in some patients, despite the vaccine’s overall safety [15]. Other research has linked the pandemic to an increase in Graves’ disease (GD), particularly among female smokers, suggesting immune system hyperactivation. The persistence of this trend remains uncertain [16].

In recent years, artificial intelligence (AI) and machine learning (ML) have demonstrated significant potential to revolutionize medical diagnostics. While the literature on their application to identify relevant biomarkers for specific pathologies remains limited, AI is anticipated to play a pivotal role in pathology workflows. Algorithms based on imaging and computational pathology enhance diagnostic accuracy, particularly in complex cases such as cancer, and automate tasks like immunohistochemical biomarker evaluation [17,18,19]. AI is also transforming pathology education through interactive training environments. However, its integration raises ethical concerns, including patient privacy and consent. Effective collaboration between pathologists and AI technologies is essential to ensure that these tools augment, rather than replace, professional expertise.

This study aims to illustrate how the integration of ML algorithms in emergency services can rapidly identify critical biomarkers for vital prognosis, with a particular focus on COVID-19 patients. By analyzing extensive epidemiological, clinical, analytical, and radiological data, the proposed approach seeks to enhance the identification of severe disease patterns.

## 2. Results

This section presents the results obtained from patient records used for training and validation, focusing on the prediction of mortality in COVID-19 patients, as well as the identification of the factors with the greatest influence on mortality. The performance of the proposed system has been compared with various ML classification methods widely recognized in the scientific community. The most significant variables in this prediction were also analyzed, employing widely recognized standard parameters as detailed in Table 1 and Table 2. The results, presented in Table 1, indicate that the Random Forest (RF) model developed in this study outperformed the other methods in terms of performance, achieving an accuracy of nearly 93%. Specifically, it demonstrated 5.44% higher accuracy than the k-Nearest Neighbors (KNN) algorithm and 15.56% higher than the Gaussian Naive Bayes (GNB). In contrast, the Bayesian linear discriminant analysis (BLDA), Decision Trees (DT), and Support Vector Machines (SVM) methods recorded the lowest accuracy rates among those analyzed.

Additional metrics were assessed, including the Youden’s Index (DYI), Matthews Correlation Coefficient (MCC), Kappa Index, and Area Under the Curve (AUC). The MCC stands out as a robust statistical measure, as its score reflects the effectiveness of predictions considering all four categories of the confusion matrix (true negatives, false negatives, true positives, and false positives), as well as the balanced distribution of positive and negative instances in the dataset. As indicated in Table 2, the RF model presented in this study achieved an MCC close to 1, demonstrating superior accuracy in mortality prediction compared to other methods. Additionally, the Kappa Index, analyzed in Table 2, confirmed that the RF system significantly outperformed both KNN and GNB.

**Figure 1:**
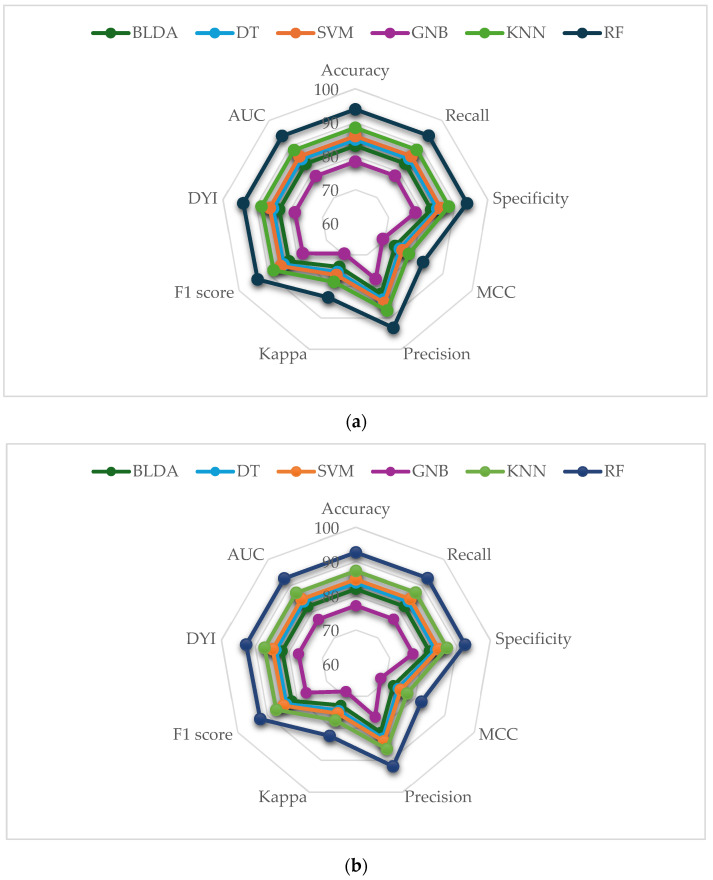
(**a**) illustrates the results of the training phase using a radar plot to display all the parameters analyzed in the study, where a larger area indicates higher predictive accuracy. (**b**) presents the results of the testing phase, also in radar plot format, allowing for a direct comparison between the algorithms evaluated.

To enhance clarity in the representation of the results, the measurements of the training and test datasets (Figure 1) were grouped using radar charts. In this format, a complete circle on the grid indicates ideal performance across all metrics. It is crucial that the test set results are consistent with those of the training set, as significant differences could indicate overfitting.

In the analysis performed, the training data consistently achieved high scores across all metrics, while the test data also obtained solid results, though slightly lower, demonstrating the absence of overfitting. As shown in Figure 1, the proposed RF model excelled in both phases (training and testing) with balanced performance. On the other hand, while KNN and GNB showed similar results, the SVM algorithm demonstrated more limited predictive capacity.

**Figure 2:**
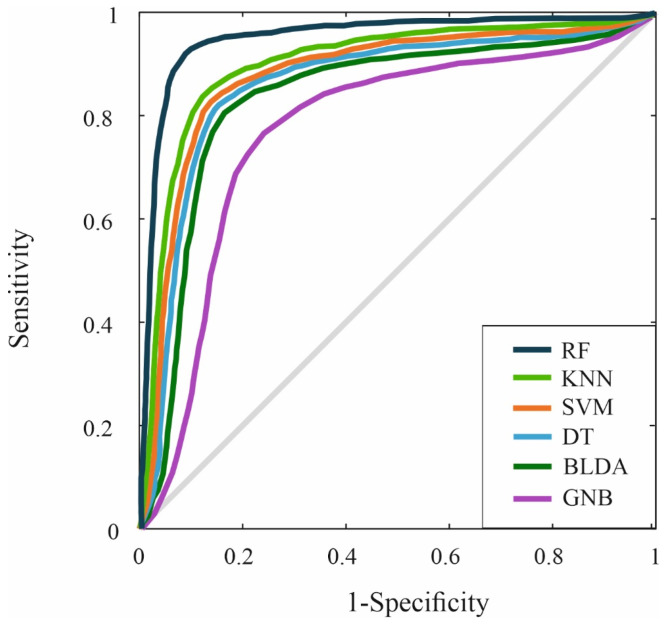
This figure presents the ROC curves for the six ML algorithms that were evaluated.

The Receiver Operating Characteristic (ROC) curve was also generated to compare the performance of the proposed system with other ML methods. The results of different systems in predicting mortality variables are presented in Figure 2. In this figure, the proposed RF model achieved an AUC of 0.93, outperforming the KNN method, which obtained an AUC of 0.87, the closest in performance.

Despite optimization of the KNN model, the results consistently demonstrated that the RF model provides superior performance in key metrics such as accuracy, recall, F1 score, and AUC-ROC. This is attributed to RF’s inherent ability to handle complex interactions between features and its boosting approach, which iteratively adjusts errors, something that KNN, based on the bagging method, does not achieve in the same manner.

Furthermore, the proposed RF method offers several key advantages in ML. It is a robust and flexible model that combines multiple decision trees to improve accuracy and reduce the risk of overfitting. Through randomization in both sample selection and feature splitting for nodes, RF performs well with noisy data and irrelevant variables. Additionally, it is effective for classification problems and can handle large datasets with many features without requiring normalization. It also provides variable importance metrics, enhancing model interpretability.

**Figure 3:**
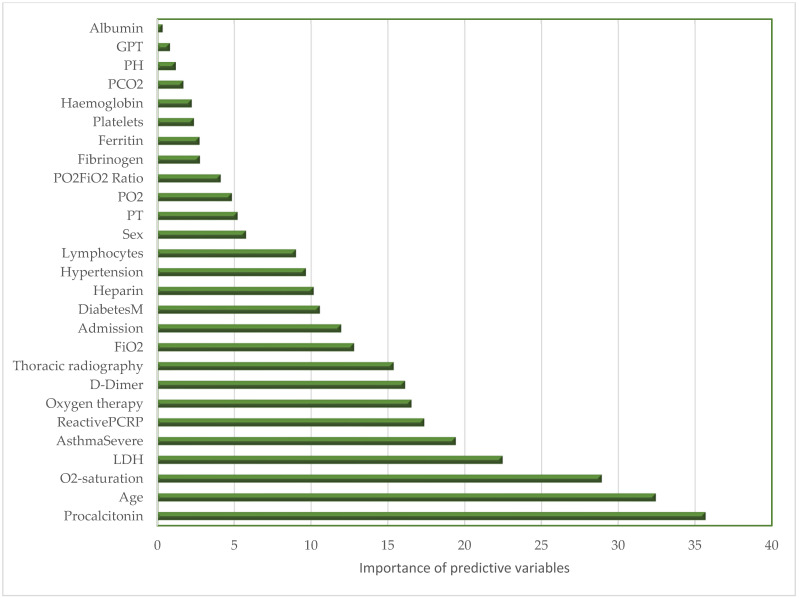
The figure presents a histogram showcasing the key parameters that contribute to the prediction of mortality in emergency COVID-19 patients.

On the other hand, the RF model assigned weights to the most relevant variables for predicting mortality. The most significant variables included elevated levels of procalcitonin (PCT), patient age, and initial oxygen saturation measured in the emergency department (Figure 3). Other important factors identified were LDH, CRP, chest X-ray infiltrates, and D-dimer levels. Elevated PCT levels are associated with severe inflammatory responses and secondary bacterial infections, often signaling complications such as sepsis or organ dysfunction. The age in our study conducted during the first wave behaved as a risk factor associated with mortality, although it is true that some authors have described differences in severity with the Omicron variant. Reduced oxygen saturation reflects hypoxemia, a critical marker of respiratory failure. Elevated LDH levels indicate a certain degree of tissue damage and are associated with increased lactate production, which often occurs in hypoxemic conditions, although LDH levels may also rise in other pathophysiological processes. Patients with severe asthma face an increased risk of respiratory complications due to pre-existing airway inflammation. Elevated CRP (C-reactive protein) levels indicate a state of hyperinflammation, which may result from inflammatory, infectious, autoimmune, or other processes, while the need for oxygen therapy signifies advanced disease and significant pulmonary impairment. Together, these variables not only highlight key pathophysiological processes in COVID-19 but also enable effective risk stratification and timely clinical interventions [20,21,22].

The system also highlighted the importance of oxygen therapy and the presence of comorbidities. Biochemical values such as ALT and albumin levels had a moderate impact on the prediction.

The system has been validated with an external database from the General University Hospital of Valencia to verify the proposed method. During the same time period, consecutive patients who tested positive for SARS-CoV-2 via PCR, aged over 18, and presenting with symptoms associated with COVID-19 were included. This new dataset comprised 200 patients. As can be seen in Table 3 and Table 4, the RF algorithm maintains a similar behavior to that produced with the Hospital Virgen de la Luz data. Specifically, RF demonstrated consistent performance metrics, including accuracy, precision, specificity, and sensitivity.

On the other hand, other ML methods, such as DT, SVM, and KNN, exhibited a decline in accuracy and failed to match the performance of the RF algorithm in this external validation. These results confirm the robustness of the RF model and its capacity to generalize effectively across different datasets.

With these findings, the proposed system proves to be highly accurate in identifying critical biomarkers and predicting mortality in COVID-19 patients. This reinforces its utility as a practical tool that can assist physicians in emergency departments to optimize patient management and resource allocation during emerging pandemics.

## 3. Discussion

Several recent studies highlight the impact of AI in biomarker discovery, particularly in the fields of oncology and personalized medicine. Advanced AI tools, such as deep learning and spatial biology, are being employed to identify key biomarkers that predict tumor responses to treatments, including immunotherapies. For instance, these models analyze complex tissue structures and cellular interactions to enhance diagnosis and personalize treatments, excelling in recognizing biomarkers such as PD-L1 expression, which can predict the effectiveness of immunological therapies in patients [23,24].

In this context, AI also facilitates analyses that combine genomic and epigenomic data to identify gene expression patterns and deoxyribonucleic acid (DNA) methylation changes directly linked to cancer [25,26], advancing toward highly personalized treatment strategies based on the molecular profile of each patient.

Another application of AI is in the identification of imaging biomarkers in the field of pathology, where deep neural networks are capable of analyzing radiological and histological data to improve colorectal cancer detection rates [27,28] or provide more precise diagnoses of precancerous cervical lesions [29,30]. These tools help identify factors that predict metastasis or recurrence, enabling the design of individualized treatment plans. AI is accelerating these processes by automating and improving biomarker validation studies, streamlining the drug development pipeline.

These advances demonstrate how AI could revolutionize biomarker discovery, speeding up the development of more precise and personalized medical interventions.

In our study, we used the COVID-19 pandemic as a case example. The most influential variable identified was PCT, followed by LDH, CRP, and D-dimer. During a pandemic, it is crucial to quickly and accurately identify the biomarkers that have the most impact on mortality in order to combat the disease effectively. AI could provide this response right from the hospital emergency services.

To date, studies have investigated the relationship between PCT and bacterial co-infection in COVID-19 patients with viral pneumonia [21,31], but there are no studies linking this biomarker as a standalone prognostic value. It was not until late 2023 that PCT began to be interpreted as an inflammation marker in severe disease [32,33].

PCT is a serum polypeptide found in minimal amounts (0.5 ng/mL) in plasma and can rise within hours in severe bacterial infections. Its synthesis is triggered by bacterial endotoxins and inflammatory cytokines, primarily interleukin (IL)-1beta, IL-6, and tumor necrosis factor-alpha [34]. Under normal conditions, it is primarily synthesized by thyroid C-cells. We believe that AI could have led to these results much earlier.

Our study also highlighted the importance of the LDH variable. LDH is an enzyme involved in lactate production, a byproduct generated when an organ experiences oxygen deprivation, as discovered later [35]. We believe that with the use of AI, we could have linked it to acute respiratory distress syndrome (ARDS) much earlier, closely monitoring patients at risk of poor outcomes, optimizing detection, and conducting early studies to achieve curative treatment. In an emerging pandemic, this early identification could change the natural history of a disease.

It is true that these markers may suggest bacterial infections of any other origin or state of tissue hypoxia that could result from other types of pathologies. The most valuable feature we aim to leverage from AI is its ability to rapidly analyze large volumes of data, providing an initial approximation of the most significant biomarkers. These biomarkers might already be known or associated with a specific pathology; however, given that medicine has very few disease-specific markers, identifying their potential relevance to another disease could prove highly significant. This becomes even more critical in the context of an emerging disease or a known pathology exhibiting atypical behavior and approached directly from the hospital emergency department. Subsequent studies will be conducted to achieve a more precise understanding of the disease; however, AI will assist in the initial approach.

Early identification of predictive variables in any emerging disease is crucial to saving lives, as it allows for the identification of patients at higher risk of complications before they manifest. Early identification facilitates the implementation of timely interventions, such as specific treatments, intensive monitoring, or preventive measures, which can make the difference between recovery and fatal outcomes. Additionally, understanding these variables in the emergency setting helps optimize medical resources, prioritizing care for those who need it most, and contributes to the development of evidence-based public health strategies. In emerging diseases, where time is critical and initial knowledge is limited, this predictive capability can significantly reduce mortality and mitigate the overall impact of the disease [36,37,38].

Our findings enhance the understanding of AI’s role in elucidating pathogenesis and assisting in the prediction and management of severe cases of a new disease.

On the other hand, the RF algorithm is a robust and widely used technique in supervised ML, owing to its high accuracy, generalization capability, and resistance to overfitting. RF combines multiple independent decision trees through a bagging process, which helps reduce model variance and improve stability. This strategy not only reduces the risk of overfitting but also provides greater generalization ability compared to individual decision trees [39,40].

One of the main advantages of RF is its ability to handle high-dimensional data and work effectively with missing or imbalanced data, making it well-suited for classification problems. Additionally, the model can capture nonlinear and complex relationships between variables without the need for parametric adjustments [40,41,42].

Another relevant benefit is model interpretability through feature importance estimation, an integrated function in RF that allows the identification of variables that contribute the most to predictions, which is valuable in applications where interpretability is crucial. Finally, the inherently parallel design of RF enables computationally efficient implementation, making it feasible to apply to large data volumes and tasks where precision and scalability are essential [40].

The results presented support the robustness and generalization capability of the proposed system, demonstrating its effectiveness for application in diverse clinical contexts without compromising accuracy or reliability. Its implementation in an additional hospital not only validates the model in an external setting but also underscores its applicability in real-world scenarios, enhancing the identification of critical biomarkers for the vital prognosis of COVID-19 patients.

## 4. Materials and Methods

### 4.1. Patients

The study took place at the Virgen de la Luz Hospital, the primary healthcare facility for the metropolitan area of Cuenca in Castilla-La Mancha, Spain. Between 2 March and 30 April 2020, more than 13,000 individuals visited the emergency department with symptoms indicative of COVID-19. Among these, 708 cases were selected from patients who tested positive for SARS-CoV-2 through molecular detection of the virus using transcription-mediated amplification (TMA) technology with Panther equipment on nasopharyngeal swabs and were aged over 18 years with symptoms associated with COVID-19. Patients were excluded if they presented outside this period, were minors, or could not undergo PCR testing for various reasons, including patient refusal, anatomical nasopharyngeal abnormalities that prevented proper sample collection, voluntary discharge requests, or discharge prior to testing.

This observational cross-sectional study was based on data gathered from a review of medical records from the emergency department. In total, 89 variables were collected: death or discharge, sore throat, dyspnea, diarrhea, fever, chest pain, asthenia, cough, headache, anosmia, myalgia, ageusia, Glasgow Coma Scale score, quick Sequential (qSOFA) score, PO_2_/FiO_2_ ratio, pH, partial pressure of oxygen (PO_2_), partial pressure of carbon dioxide (PCO_2_), pharmacological immunosuppression, institutionalization, smoking, vascular disease, active cancer, hypertension, obesity, chronic kidney disease, dyslipidemia, diabetes mellitus, chronic obstructive pulmonary disease (COPD), severe asthma, liver disease, thromboembolic disease, nationality, age, medical record number, sex, respiratory rate, oxygen saturation, systolic and diastolic blood pressure, fraction of inspired oxygen (FiO_2_), heart rate, body temperature, thoracic computed tomography (CT) findings, emergency department chest X-ray showing bilateral or unilateral infiltrates, total proteins, creatinine, albumin, troponin, leukocyte count, D-dimer, hemoglobin, lymphocyte count, platelet count, ferritin, prothrombin time (PT), CRP, LDH, alanine transaminase (ALT), PCT, antibiotics, immunomodulators (tocilizumab, cyclosporine, anakinra, baricitinib), antivirals (lopinavir/ritonavir, emtricitabine/tenofovir disoproxil, darunavir/cobicistat), corticosteroids (dexamethasone or methylprednisolone), hydroxychloroquine, low molecular weight heparin (LMWH), bronchodilators, oxygen therapy, dexamethasone (including dose), LMWH, methylprednisolone (including dose), duration of illness, hospitalization dates, and duration.

The study protocol was approved by the Clinical Research Ethics Committee of the Hospital Virgen de la Luz. All principles of the Declaration of Helsinki and the Spanish Data Protection Act 15/1999 were strictly observed, ensuring the anonymity of the patients. The physicians responsible for data collection were distinct from those who performed the subsequent analysis.

### 4.2. Artificial Intelligence Method

The integration of AI is transforming medicine by offering advanced solutions for clinical data analysis, early diagnosis, and the development of personalized treatments. With the capacity to process large volumes of information—such as medical imaging, genomic data, and health records—it enhances precision and accelerates clinical decision-making [43]. AI-based tools, including deep neural networks and ML models, have demonstrated their effectiveness in detecting complex diseases, identifying digital biomarkers, and stratifying patient risk [43,44,45,46]. AI not only enhances diagnostic accuracy but also enables the prediction of disease progression and the adaptation of therapeutic interventions to the individual characteristics of each patient, paving the way for more personalized, preventive, and precise medicine [47,48,49,50].

In this analysis, the RF algorithm was applied, an ensemble method that leverages the bagging aggregation approach to construct multiple decision trees independently, thereby reducing variance and improving model robustness. RF employs bootstrapping, or sampling with replacement, from the training data to generate a collection of independent trees, where each tree is trained on a random subset of the original dataset. Additionally, at each tree node, a random subset of features is selected instead of considering all variables. This introduces an additional source of randomness, reduces inter-tree correlation, and enhances the ensemble’s predictive accuracy [43,51,52].

During the training process, each tree independently makes decisions, and the results are combined using a majority-voting scheme for classification. Feature importance is assessed by analyzing the decrease in accuracy or the Gini index when the values of a feature are randomly permuted. This enables the identification of the most relevant variables for the model. Finally, model performance was evaluated using specific metrics such as accuracy, sensitivity, specificity, and the AUC, validating its predictive capacity and generalization to unseen data.

In RF, an ensemble of decision tree {*T*_1_, *T*_2_, …, *T_m_*} is constructed using the bagging approach. To build each tree *T_i_*, the following steps are performed:

- Bootstrapping (Sampling with Replacement): From a training dataset containing n observations and pp features, a subset *D_i_* is generated by randomly selecting n samples with replacement from the original dataset. This technique allows certain data points to appear multiple times in *D_i_*, while others may not appear at all.
- Random Feature Selection: At each node of each tree, instead of evaluating all pp features, a random subset of kk features is selected, where *k* = $\sqrt{p}$. This reduces correlation between individual trees, enhancing the model’s generalization capability.
- Node Splitting Criterion: Each node is split based on an impurity reduction criterion, such as entropy or the Gini index, in classification tasks. In this study, the Gini index was used. The impurity GG of a node with class proportions *p_k_* is defined as:
  (1) $$G=1-\sum_{k=1}^{K}p_{k}^{2}$$
- Tree Aggregation: Once the trees are trained, RF predictions are obtained through aggregation. For a set of tree {*T*_1_, *T*_2_, …, *T_m_*}, the final prediction $\hat{y}$ is determined by majority voting:
  (2) $$\hat{y}=mode\{T_{1}\left(x\right),\;T_{2}\left(x\right),\;\ldots ,\;T_{m}\left(x\right)\}$$

- Feature Importance: The importance of each feature is measured by evaluating the change in the splitting criterion when the feature is randomly permuted in the dataset. For Gini index-based importance, a feature is considered important if permuting it increases node impurity across the trees.
- Model Evaluation: The model’s performance was assessed using metrics such as accuracy, sensitivity, specificity, and AUC in classification problems or mean squared error (MSE) in regression tasks.

**Figure 4:**
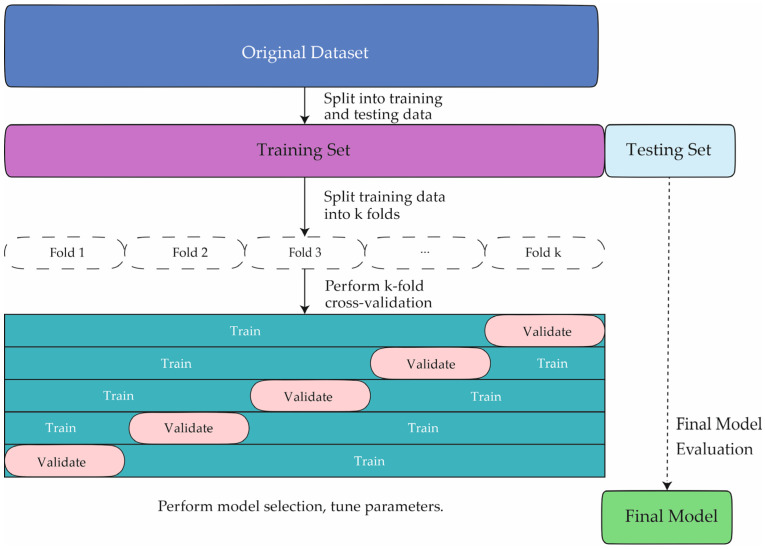
The figure illustrates the learning and validation stages employed in this study.

In this study, the proposed method underwent comprehensive evaluation by comparison with various ML techniques for classifying COVID-19 patients. Algorithms included in the comparative analysis were GNB [43], KNN [43], BLDA [43], SVM [43], and DT [43], along with the novel RF system developed in this study. The implementation and evaluation of the models were performed using MATLAB’s Statistics and Machine Learning Toolbox (version 2024a). To mitigate overfitting, a five-fold cross-validation strategy was employed. Data were split into two subsets, with 70% allocated for training and 30% for testing, ensuring independence between patient groups in each subset. Figure 4 schematically illustrates the study workflow, starting with patient selection and database creation, followed by the training and validation phases of the ML models.

The advantages of the various ML methods used in the article are outlined below. SVM is a classification-focused algorithm designed to find an optimal hyperplane in a higher-dimensional space, maximizing the margin between different classes. Additionally, SVM effectively handles non-linear data through the use of the “kernel trick”, which transforms the data into a more manageable space [43,53]. BLDA extends the approach of Linear Discriminant Analysis (LDA) by incorporating additional probabilistic assumptions. This method assumes a multivariate normal distribution within each class and applies Bayesian methodologies, making it particularly valuable in scenarios where classes exhibit distinct distributions or variances [43,54]. DT are predictive models structured as trees, comprising a root node, internal nodes, and leaf nodes. The depth of the tree directly impacts its generalization capabilities, and pruning techniques are employed to mitigate overfitting. The construction process involves iteratively selecting features to partition the data, aiming to maximize homogeneity at each split [43,55]. GNB is a variant of the Naive Bayes model that assumes a Gaussian distribution for input features. Commonly used for classification tasks, GNB requires a labeled training dataset. Parameters of the Gaussian distribution are calculated for each class, and classification is performed using Bayes’ rule, providing a probabilistic estimation [43,56]. Finally, KNN is a supervised learning algorithm that relies on the majority vote of the k nearest neighbors for classification. It depends on a labeled training dataset, employing a selected distance metric and a specified value of k. The classification process determines the label for a new point by voting among its k nearest neighbors [43,57].

The parameters of the models were optimized with a Bayesian approach. It generates a short sequence of simulated experiments with different combinations of the hyperparameters, keeping the values that present the best AUC and balanced accuracy. The configurations employed in this study are shown in Table 5.

## 5. Conclusions

The proposed algorithm has proven to be a pivotal tool in the discovery of biomarkers for emerging diseases in emergency departments, accelerating the analysis of large datasets and enabling the identification of critical factors for prognosis and treatment to guide personalized medicine. In the context of pandemics, implementing AI-based systems could facilitate the earlier identification of key biomarkers. In our study, the importance of PCT and LDH was demonstrated for prioritizing interventions in patients at vital risk. The proposed RF algorithm excels in analyzing complex patterns, integrating genomic and epigenomic data, and optimizing medical resources, contributing to the design of personalized strategies that enhance diagnostic precision and the management of severe cases. This technology not only accelerates biomarker validation but also fosters the development of innovative and tailored treatments, underscoring its significance in transforming medical responses to emerging pathologies and substantially reducing mortality.

## Figures and Tables

**Table 1:** The table displays the average values and standard deviations of specificity, recall, MCC, AUC, and F1 score.

	Recall	Specificity	MCC	AUC1	F1 Score
SVM	84.85 ± 0.87	84.65 ± 0.85	75.20 ± 0.82	0.85 ± 0.02	84.49 ± 0.88
BLDA	82.03 ± 0.96	81.83 ± 1.03	72.70 ± 0.85	0.82 ± 0.02	81.68 ± 1.01
DT	83.84 ± 0.91	83.64 ± 0.93	74.31 ± 0.92	0.84 ± 0.02	83.49 ± 0.93
GNB	77.16 ± 1.08	76.98 ± 1.10	68.39 ± 1.05	0.77 ± 0.02	76.84 ± 1.04
KNN	87.28 ± 0.74	87.07 ± 0.75	77.35 ± 0.78	0.87 ± 0.01	86.91 ± 0.73
RF	92.72 ± 0.51	92.50 ± 0.48	82.18 ± 0.45	0.93 ± 0.01	92.34 ± 0.49

**Table 2:** The table presents the average values and standard deviations of accuracy, precision, Kappa, and DYI.

	Accuracy	Precision	Kappa	DYI
SVM	84.75 ± 0.83	84.14 ± 0.84	75.45 ± 0.81	84.75 ± 0.82
BLDA	81.93 ± 0.99	81.35 ± 0.98	72.94 ± 0.95	81.93 ± 1.02
DT	83.74 ± 0.92	83.15 ± 0.90	74.55 ± 0.89	83.74 ± 0.91
GNB	77.07 ± 1.05	76.52 ± 1.03	68.61 ± 1.02	77.07 ± 1.06
KNN	87.17 ± 0.76	86.55 ± 0.74	77.61 ± 0.76	87.17 ± 0.75
RF	92.61 ± 0.49	91.95 ± 0.48	82.45 ± 0.46	92.61 ± 0.48

**Table 3:** The table displays the average values and standard deviations of specificity, recall, MCC, AUC, and F1 score for external validation.

	Recall	Specificity	MCC	AUC1	F1 Score
SVM	82.95 ± 0.82	82.67 ± 0.80	73.51 ± 0.83	0.82 ± 0.02	82.76 ± 0.83
BLDA	79.83 ± 1.02	79.89 ± 1.05	71.02 ± 0.91	0.79 ± 0.02	79.76 ± 1.03
DT	81.56 ± 0.96	81.48 ± 0.97	69.86 ± 0.95	0.81 ± 0.02	81.37 ± 0.94
GNB	74.99 ± 1.09	74.87 ± 1.12	66.59 ± 1.07	0.74 ± 0.02	74.78 ± 1.06
KNN	85.23 ± 0.76	85.19 ± 0.76	76.52 ± 0.72	0.85 ± 0.01	85.31 ± 0.78
RF	91.84 ± 0.52	91.35 ± 0.51	81.09 ± 0.49	0.91 ± 0.01	91.57 ± 0.51

**Table 4:** The table presents the average values and standard deviations of accuracy, precision, Kappa, and DYI for external validation.

	Accuracy	Precision	Kappa	DYI
SVM	82.89 ± 0.89	82.67 ± 0.91	73.58 ± 0.88	82.54 ± 0.89
BLDA	79.90 ± 1.02	79.96 ± 1.03	71.01 ± 0.97	79.87 ± 1.04
DT	81.39 ± 0.96	81.42 ± 0.94	70.13 ± 0.93	81.37 ± 0.95
GNB	75.06 ± 1.08	75.48 ± 1.06	66.53 ± 1.04	75.02 ± 1.07
KNN	85.34 ± 0.78	85.27 ± 0.79	75.93 ± 0.77	85.22 ± 0.78
RF	91.75 ± 0.52	91.47 ± 0.51	81.34 ± 0.51	91.57 ± 0.52

**Table 5:** Main hyperparameters of the machine learning algorithms evaluated in the study.

Method	Parameters
SVM	Kernel function: Gaussian Sigma = 0.5 C = 1.0 Numerical tolerance = 0.001 Iteration limit = 100
DT	Minimum number of instances in leaves = 4 Minimum number of instances in internal nodes = 6 Maximum depth = 100
BLDA	Kernel: Bayesian
GNB	Usekernel: False fL = 0 Adjust = 0
KNN	Number of neighbours = 20 Distance metric: Euclidean Weight: Uniform
RF	Number of estimators: 120, Maximun_depth: 20, Minimum_samples_split: 10, Minimum_samples_leaf: 4, Maximun _features: ‘sqrt’

## Data Availability

The datasets used and/or analyzed during the present study are available from the corresponding author upon reasonable request.
